# Revisit the Candidacy of Brain Cell Types as the Cell(s) of Origin for Human High-Grade Glioma

**DOI:** 10.3389/fnmol.2018.00048

**Published:** 2018-02-21

**Authors:** Fangjie Shao, Chong Liu

**Affiliations:** Department of Pathology and Pathophysiology, Zhejiang University School of Medicine, Hangzhou, China

**Keywords:** cell of origin, high-grade glioma, glioblastoma, adult neural stem cells (NSCs), oligodendrocyte precursor cells (OPC), genetically engineered mouse models (GEMMs), lineage tracing

## Abstract

High-grade glioma, particularly, glioblastoma, is the most aggressive cancer of the central nervous system (CNS) in adults. Due to its heterogeneous nature, glioblastoma almost inevitably relapses after surgical resection and radio-/chemotherapy, and is thus highly lethal and associated with a dismal prognosis. Identifying the cell of origin has been considered an important aspect in understanding tumor heterogeneity, thereby holding great promise in designing novel therapeutic strategies for glioblastoma. Taking advantage of genetic lineage-tracing techniques, performed mainly on genetically engineered mouse models (GEMMs), multiple cell types in the CNS have been suggested as potential cells of origin for glioblastoma, among which adult neural stem cells (NSCs) and oligodendrocyte precursor cells (OPCs) are the major candidates. However, it remains highly debated whether these cell types are equally capable of transforming in patients, given that in the human brain, some cell types divide so slowly, therefore may never have a chance to transform. With the recent advances in studying adult NSCs and OPCs, particularly from the perspective of comparative biology, we now realize that notable differences exist among mammalian species. These differences have critical impacts on shaping our understanding of the cell of origin of glioma in humans. In this perspective, we update the current progress in this field and clarify some misconceptions with inputs from important findings about the biology of adult NSCs and OPCs. We propose to re-evaluate the cellular origin candidacy of these cells, with an emphasis on comparative studies between animal models and humans.

## Introduction

Adult gliomas are the most common cancers of the central nervous system (CNS) (Louis, 2006; Perry and Wesseling, 2016). Despite many years of efforts in both basic research and clinical practice, the prognosis of malignant gliomas, particularly the most advanced one, glioblastoma multiforme (GBM), remains dismal. This lack of progress is largely associated with high inter- and intra-tumoral heterogeneity. Tumor tissues from not only different patients, but also from the same ones, can be stratified into distinct morphopathological groups or molecular subtypes (Verhaak et al., 2010; Snuderl et al., 2011; Brennan et al., 2013; Kim J. et al., 2015; Wang et al., 2016, 2017). Such heterogeneity is generally considered as the main reason for drug resistance and high recurrence rate during treatment.

A cell of origin is the normal progenitor from which all the neoplastic cells of a given type of cancer develop (Visvader, 2011; Chaffer and Weinberg, 2015). Identification of the cell of origin can give critical insights into the principles dictating tumor heterogeneity, therefore holding great promise in understanding the cancer etiology, and facilitating the design of effective therapeutic strategies. In this *Perspective*, we review the current progress in the research of the cell of origin of glioma. Together with new findings in NSCs and OPCs from both rodents and large-brained mammals including humans, we propose to carefully re-evaluate the candidacy of several popular cell types that have been believed as the potential cells of origin of glioma in humans.

## CNS Cell Types Relevant to Glioma Etiology: Their Lineage Relationship and Some Important Updates

Knowing the properties of neural cell types and their lineage relationship will help understanding their potential contributions to the etiology of human glioma. Neural cells in the adult CNS are grossly classified as neurons, astrocytes, oligodendrocyte precursor cells (OPCs), and oligodendrocytes. In addition to these lineage-committed progenitor and mature cells, specialized stem cells, termed adult neural stem cells (NSCs) exist within restricted regions such as the subventricular zone (SVZ) next to the lateral ventricle, and the subgranular zone (SGZ) of the hippocampus (Ming and Song, 2011), in the adult brain. Both SVZ adult NSCs and OPCs have been implicated as the major candidates for glioma cell of origin, therefore, deserving a little more discussion.

### Adult Neural Stem Cells (NSCs)

**FIGURE 1:**
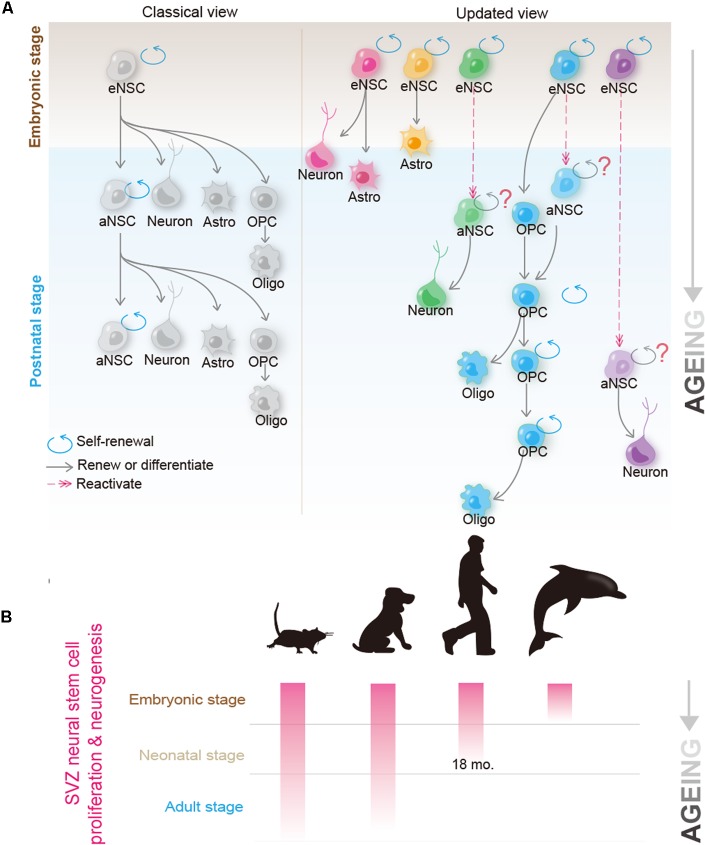
Recent progress in the biology of neural stem cells (NSCs) and oligodendrocyte precursor cells (OPCs) provides new insights into their candidacy as the cell of origin for human glioblastoma. **(A)** Classical (left) and the updated (right) view of the NSC behavior in the brain. In the classical view, it was believed that a single NSC can repeatedly self-renew for many generations and give rise to new NSCs; at the same time, it possesses the potential to differentiate into neurons, astrocyte and OPCs. OPCs can further differentiate into mature oligodendrocytes. Recent studies using mouse models suggest that adult B1 cells (adult form of NSCs) are derived from eNSCs that actively proliferate at ∼E14.5. These embryonic NSCs remain quiescent until they are reactivated at the adult stage. Though as a whole population, adult NSCs continuously proliferate and give rise to olfactory bulb (OB) neurons and glial cells, they are extremely heterogeneous at the single cell level. Clonal analysis revealed that a single adult NSC can either give rise to OB neurons or glial cells (such as astrocytes and oligodendrocytes), but rarely to both cell types. Furthermore, many, if not all, adult NSCs cannot bud off OB neurons and simultaneously self-renew, raising the question of whether adult NSCs conform to the hardwired definition of self-renewable tissue stem cells (Fuentealba et al., 2015; Furutachi et al., 2015). On the other hand, despite OPCs originally being derived from NSCs during early development, and adult NSCs contributing to the OPC pool to some extent, in the normal brain, most adult OPCs are generated from the locally resident OPCs. Clonal analysis further revealed that adult OPCs can self-renew continuously (Garcia-Marques et al., 2014; Yeung et al., 2014). **(B)** Neurogenic and proliferative activities of SVZ NSCs in rodent, dog, human, and dolphin. Please note that neurogenic activity disappears in human at ∼18 months, and is completely absent in dolphin postnatally; however, both species can suffer from GBM at adulthood. Kindly refer to the main text for further details.

Adult NSCs (also termed B1 cells), which were best studied in rodents, have been generally believed to be able to persistently self-renew, and give rise to multiple neuronal and glial cell types (Alvarez-Buylla et al., 2001). Recent progresses in NSC biology, however, may suggest a quite different scenario. By using a temporal Histone 2B-EGFP marking system or barcoded retroviral labeling-based clonal analysis, two groups independently reported that postnatal B1 cells are derived from embryonic NSCs that divide during mid-fetal development and then remain quiescent until they reactivate, thus generating progeny in the postnatal brain (Fuentealba et al., 2015; Furutachi et al., 2015). Surprisingly, clonal analysis unraveled that postnatally, a single B1 cell neither divides repeatedly to produce generations of olfactory bulb (OB) neurons, nor gives rise to cortical glial cells and OB neurons simultaneously, raising an interesting possibility that adult NSCs may not systematically self-renew (Fuentealba et al., 2015) (see also **Figure 1A**). Therefore, although adult NSCs exhibit remarkable self-renewal potential and differentiation plasticity in culture (Doetsch et al., 1999; Codega et al., 2014), it remains highly debated whether, in the brain, they conform to the hardwired definition of tissue stem cells, as seen in the case of hematopoietic or intestinal stem cells (Batlle and Clevers, 2017).

### Oligodendrocyte Precursor Cells (OPCs)

Oligodendrocyte precursor cells were initially thought to function solely as transient forms of glial progenitors, to generate mature oligodendrocytes. Nevertheless, recent studies show that even though some OPCs indeed differentiate, many of them retain the ability to self-renew (Nishiyama et al., 2009; Vigano and Dimou, 2016) (see also **Figure 1A**). By using a sensitive DNA-labeling approach to mark cells undergoing proliferation, Yeung et al. (2014) showed that all OPCs in the adult mouse brain were dividing. Strikingly, Garcia-Marques et al. (2014 observed that at the clonal level, a single OPC could give rise to up to 400 cells in the adult mouse brain, therefore unequivocally demonstrating that OPCs are a *bona fide* self-renewable cell population *in vivo*. Given that OPCs make up 5–10% of all cells in the brain (Dawson et al., 2003), using the absolute number as the criteria, OPCs should be viewed as the largest proliferation pool in the mammalian brain. In addition to self-renewal, OPCs have been reported to exhibit some lineage plasticity. Despite being a matter of intensive debate, OPCs were shown to be able to differentiate into astrocytes and/or neurons *in vivo* (Rivers et al., 2008; Zhu et al., 2008, 2011; Richardson et al., 2011), and can be reprogrammed into the NSC-like status *in vitro* (Kondo and Raff, 2000), thus resembling NSCs in ways stronger than those previously considered (Richardson et al., 2011).

## The Research Progress of Glioma Cellular Origins

### NSCs as the Cell of Origin: Evidence and Concerns

Adult NSCs have been widely viewed as the most possible cell of origin for high-grade glioma, given their prominent property to self-renew, and the remarkable plasticity to differentiate into multiple neural cell types (Doetsch et al., 1999; Alvarez-Buylla et al., 2001; Stiles and Rowitch, 2008). In addition, cancer stem cells (CSCs) isolated from human GBMs share many markers normally expressed by NSCs (such as Nestin, GFAP, CD133, and Sox2), and are able to form renewable NSC-like spheres in culture (Singh et al., 2004; Bao et al., 2006). Furthermore, mouse and human NSCs can be transformed *in vitro*; they gain the capacity to develop into gliomas after implantation into host mice (Bachoo et al., 2002; Duan et al., 2015). Importantly, delivery of DNA or viral vehicles into the embryonic, neonatal, or adult SVZ (the brain structure where NSCs reside) to introduce over-expression of oncogenes and/or knockout/knockdown of tumor suppressor genes could efficiently generate high-grade glioma in mice (Alcantara Llaguno et al., 2009; Marumoto et al., 2009; Breunig et al., 2015; Zuckermann et al., 2015). Intriguingly, human glioblastomas were frequently diagnosed next to the SVZ, further supporting the possibility that they originated from NSCs (Barami et al., 2009). More direct evidence was obtained from the lineage-tracing experiment by using genetically engineered mouse models (GEMMs). Taking advantage of NSC-specific genetic tools such as hGFAP-Cre, Nestin-Cre, or Nestin-Cre^ER^, Parada and his colleagues showed that mouse NSCs are capable of transforming into high-grade gliomas after the loss of *Trp53*, *NF1* and/or *PTEN* (Zhu et al., 2005; Chen et al., 2012; Alcantara Llaguno et al., 2015).

While these multiple lines of evidence demonstrate that NSCs are capable of transforming into malignancy, several important issues should be understood. Firstly, as already mentioned, recent findings about NSC biology challenges the concept that a single SVZ aNSC can repeatedly self-renew, therefore greatly decreasing the possibility for an aNSC to accumulate mutations, as previously assumed. Secondly, the stem cell feature of CSCs need not necessarily be inherited from tissue stem cells; it can also be regained through the de-differentiation of lineage-committed progenitors or mature cells (Batlle and Clevers, 2017). Thirdly, many claimed that NSC cellular markers are not specific to NSCs. For example, the most widely used NSC marker Nestin, an intermediate filament protein expressed in radial glia and adult B1 cells, is prominently expressed in reactive astrocytes (Ernst and Christie, 2006). Although partial overlaps between brain tumor locations and the NSC niche is a good argument to support the fact that gliomas originate from adult NSCs in patients, a recent work revealed that the SVZ may merely function as a niche toward which glioma cells prefer to migrate (Qin et al., 2017).

An additional dimension of complexity comes from the nature of NSCs *per se*. As NSCs can readily differentiate into fate-committed precursors such as OPCs or mature astrocytes, it is unclear whether NSCs, after acquiring initial mutations, directly transform, or they must proceed through the status of lineage-committed cell types prior to the final transformation. In fact, by using a single-cell resolution genetic mouse model termed mosaic analysis with double markers (MADM), we have shown that introducing *p53* and *NF1* mutations into NSCs did not evidently change the proliferation rate of pre-cancerous adult NSCs, but drastically promoted the over-expansion of descendant OPCs, arguing against a direct transformation of NSCs, at least in the context of this mutation combination (Liu et al., 2011).

### OPCs as the Cell of Origin: Evidence and Some Updates

Oligodendrocyte precursor cells have been proposed as an important cell of origin for glioma since they were first identified. As already mentioned, OPCs represent the largest proliferation pool in the brain, and exhibit remarkable self-renewal capacity both *in vitro* and *in vivo*, and are therefore suitable, as cells of origin, to accumulate genetic mutations. In fact, NG2, one of the most commonly used OPC cell marker, was initially isolated from a rat glioma model (Stallcup, 1981). In addition to NG2, we and others showed that many cellular markers typically expressed in OPCs, such as Olig2, PDGFRa, and O4, were also expressed in most, if not all, human malignant gliomas (Shoshan et al., 1999; Ligon et al., 2004, 2007; Rebetz et al., 2008; Ledur et al., 2016; Shao et al., 2017). Furthermore, over-expression of the oncogenic form of EGFR (EGFRvIII) under the promoter S100b, a non-stem cell marker (Raponi et al., 2007), induced gliomas recapitulating the pathological features of human oligodendroglioma (Weiss et al., 2003; Persson et al., 2010). Moreover, overexpression of PDGF-BB alone, or when combined with p53 and Pten deactivation, was shown to be able to effectively transform rat and mouse OPCs into lower-grade oligodendrogliomas or high-grade gliomas (Assanah et al., 2006; Lindberg et al., 2009; Lei et al., 2011; Lu et al., 2016). More direct evidence to support the OPC-origin of high-grade gliomas comes from fate-mapping experiments. By using OPC-specific NG2-Cre or NG2-Cre^ERT^ transgenic mouse lines, we and others have provided convincing evidence that OPCs, after acquiring *Trp53* and *NF1* mutations, can be directly transformed into malignant gliomas resembling the proneural subtype of GBM, whenever the mutations were introduced in early or adult stage (Liu et al., 2011; Galvao et al., 2014; Alcantara Llaguno et al., 2015).

The data from our group show that OPC-like tumor cells are universally present in all human malignant gliomas, and share remarkable similarities in many aspects with their counterparts found in mouse genetic models, in which OPCs are the defined cells of origin (Ledur et al., 2016; Shao et al., 2017). These lines of evidence collectively lead to a reasonable assumption that OPCs are important glioma cells of origin in patients.

### Mature Astrocytes and Neurons as the Cells of Origin: An Unsettled Issue

Whether mature astrocytes and/or neurons are able to directly transform remains highly debated. Chow et al. (2011) utilized GFAP-Cre^ER^ to introduce Trp53, Pten and/or Rb1 mutations into astrocytes and induced high-grade astrocytomas in adult mice. Also using GFAP-Cre^ER^, Vitucci et al. (2017) observed that murine astrocytes could transform into high-grade glioma mimicking human mesenchymal, proneural, and neural GBMs. By using Cre-activatable lentiviral vehicles that encoded shRNA against Trp53 and NF1, Friedmann-Morvinski et al. (2012) reported high incidence of GBMs when they transfected such lentiviral particles into the brains of hGFAP-Cre, Synapsin I-Cre or CamK2a-Cre transgenic mice. Therefore, the authors claimed that both mature astrocytes and neurons can function as the cells of origin for GBMs through dedifferentiation. Nevertheless, as most claimed astrocyte-specific markers such as GFAP are also expressed in NSCs (Chaker et al., 2016), and those for neurons like Synapsin-1 are also expressed in OPCs [(Cahoy et al., 2008; Zhang et al., 2014) and personal observations], further validation is necessary to exclude the possibility of targeting NSCs and/or OPCs when attempting to manipulate mature astrocytes or neurons. Highly specific genetic tools are warranted to clarify this fundamental issue.

## Human Relevance: New Data and the Insight From Comparative Studies

Most of our current knowledge on glioma cell of origin was derived from the observations on animal models, mostly GEMM-based cancer models. One fundamental question we must confront is that how much of the landscape depicted thus far can be directly extrapolated to human cases. Despite the overall anatomical structures and developmental principles of the CNS being highly conserved among mammals, notable differences, particularly in the properties of adult NSCs, do exist among species. Recognizing these differences has important impacts on shaping our understandings of the glioma cell of origin in humans.

### Adult NSCs May Not Be a Major Player in the Pathogenesis of Glioblastoma in Human or Other Large-Brained Animals

Unlike in rodents, where NSCs and neural progenitors proliferate continuously to form new neurons, in large-brained mammals, such as humans, SVZ neurogenesis declines drastically during postnatal life (Lipp and Bonfanti, 2016; Paredes et al., 2016), and fully disappears at around 18 months (Sanai et al., 2011), long before high-grade gliomas are diagnosed. Consistent with this observation, by measuring the turnover rate of nuclear bomb test-derived ^14^C in genomic DNA, Bergmann et al. (2012, 2015) showed that there is virtually no postnatal neurogenesis in the human OB.

Direct evidence to support a lack of marked levels of neurogenesis or self-renewal of NSCs in the adult human SVZ comes from immunohistological studies, where proliferative cells were rarely found in the SVZ in adults (Wang et al., 2011; Dennis et al., 2016). Furthermore, the density of dividing cells in the SVZ is comparable to or even lower than that in other regions such as the corpus callosum (Shao et al. personal observations). Despite the suggestion that certain pathological conditions such as ischemic stroke may activate NSCs in the adult human brain (Jin et al., 2006; Marti-Fabregas et al., 2010), this conclusion was disproved by the ^14^C turnover assay (Huttner et al., 2014). Regardless the potential of adult NSCs to be activated *in vivo* after injury, no definitive evidence yet shows an association of human glioma pathogenesis with any pathological lesions.

Comparative studies between species provide deeper insights into questioning the relevance of adult NSCs in glioma pathogenesis (as summarized in **Figure 1B**). Unlike humans, but quite similar to rodents, dogs possess SVZ neurogenesis that persists into adulthood (Malik et al., 2012). Therefore, one may expect a much higher incidence of gliomagenesis in dogs, if adult NSCs indeed play critical roles in initiating glioma. Contrary to this speculation, epidemiological studies suggest that the incidence of spontaneous brain tumors in dogs is remarkably similar to that in humans, i.e., approximately 20 in 100,000 per year (Dobson et al., 2002; Hicks et al., 2017). On the other hand, aquatic mammals such as dolphins, which lack a functional periventricular germinal layer postnatally and any detectable dividing cells within the SVZ (Parolisi et al., 2017), can surely suffer from glioblastoma (Diaz-Delgado et al., 2015). These findings, together with those in rodent NSCs, contradict the argument that adult NSCs play major roles in initiating gliomagenesis.

### Adult OPCs May Function as an Important Cell of Origin with Strong Human Relevance

Unlike the great variations of cellular behaviors of adult NSCs, the renewal capacity of OPCs are largely conserved across species. For instance, immunohistological studies show that, although sparse, OPCs are the major cycling cells in the adult human brain (Geha et al., 2010). In line with this observation, ^14^C data revealed that gray matter oligodendrocytes do not reach a plateau until the fourth decade of life, even after which the annual turnover remains as high as 2.5% (Yeung et al., 2014). These results in collection clearly demonstrate that OPCs undergo substantial renewal in the adult human brain.

Interestingly, the proliferation rate of OPCs are significantly elevated in epileptic patients (Geha et al., 2010). As epilepsies are frequently associated with glioma patients (Iuchi et al., 2015; Englot et al., 2016), these observations raise an intriguing possibility that aberrant neuronal activity may directly contribute to OPC self-renewals and, most likely, to oncogenic transformation. This hypothesis has been recently substantiated by showing that artificially enhancing the neuronal activity in GEMMs through optogenetic approaches can stimulate the proliferation of normal resident OPCs and engrafted human GBM cells (Gibson et al., 2014; Venkatesh et al., 2015).

Therefore, although comprehensive studies are warranted to systematically characterize the relative proliferating capacities of OPCs and NSCs/NPCs in the adult human brain *in situ*, given that OPCs retain a relatively decent level of self-renewal activity, and significantly outnumber NSCs in the adult human brain, they remain a highly probable candidate for the cell of origin of human GBMs.

## The Relationship Between Cell(s) of Origin and Cancer Stem Cells (CSCs)

It should be noted that the “CSC” is a functional definition that can only be assessed by the capacity of a cancer cell to initiate new tumors. Some studies identified CSCs from the NSC-derived GBM mouse models and showed that these NSC-derived CSCs resemble normal NSCs in certain ways such as the expression of Nestin (Zheng et al., 2008; Alcantara Llaguno et al., 2009; Chen et al., 2012). However, the cells functioning as CSCs may not have to be derived and/or resemble normal NSCs. By using S100b- promoter-driven EGFRViii transgene, Persson et al. (2010) clearly showed that oligodendroglioma can be initiated from non NSCs, and the CSCs in this model can be identified and isolated based on their expression of NG2 (CSPG4), an OPC marker. We showed that CSCs derived from OPC-originated HGGs expressed NG2 as well as other OPC markers (such as PDGFRa and Olig2) and that the OPC feature is essential for the maintenance of the stemness of these CSCs (Liu et al., 2011; Ledur et al., 2016). Interestingly, OPC-originated CSCs gained the capacity to form spheres and to express Nestin. This latter observation implicates that Nestin is a marker for the stemness but not the cell identity in this particular case. In the human cases, NG2 have been used to enrich CSCs from oligodendrogliomas (Persson et al., 2010) and at least some GBMs (Persson et al., 2010; Al-Mayhani et al., 2011). Our own study showed that human primary GBM cell lines maintained under culture conditions that favor the enrichment of OPC-like tumor cells have enhanced malignancy (Ledur et al., 2016). In addition to OPCs, Schmid et al. (2016) provided the evidence that mature astrocytes could dedifferentiate into glioma CSCs upon transformation. Therefore, CSCs in gliomas can definitely be developed from the non-CSC cell types. The detailed lineage relationship between NSCs, lineage-committed progenitors, mature cells and CSCs remains to be fully elucidated in the future studies.

## The Relationship Between Cells of Origin, Tumor Subtypes and Heterogeneity

Cumulative evidence suggests that the same cell of origin can give rise to the GBMs manifesting different molecular features and that distinct types of cells of origin can evolve in parallel to give rise to tumors resembling similar molecular features (see also **Table 1**).

**Table 1:** Pathological features and molecular signatures of currently reported GEMMs for gliomas.

Putative cell of origin	Mutations	Approach	Molecular subtype	Pathology	Reference
NSC	Ras, Akt	RCAS/tv-a system	NA	GBM	Holland et al., 2000
	Ink4a, Arf, EGFR	Retrovirus	NA	High-grade gliomas	Bachoo et al., 2002
	H-Ras, AKT	Lentivirus + GFAP-Cre mice	NA	GBM	Marumoto et al., 2009
	Trp53, Nf1, and/or Pten	Adenovirus + Nestin-CreER	NA	A	Alcantara Llaguno et al., 2009
	PTEN, Trp53	Adenovirus-Cre	NA	High-grade gliomas	Jacques et al., 2010
	Ras; Erbb2; Pdgfra	Plasmid DNA + Electroporation	Proneural, Neural, Mesenchymal	AA, AO, AOA, GBM	Breunig et al., 2015
	Trp53, Pten, Nf1	CRISPR/Cas9 + Electroporation	NA	GBM	Zuckermann et al., 2015
	Trp53, NF1	hGFAP-Cre	NA	A, AA, GBM	Zhu et al., 2005
	Trp53, Pten	hGFAP-Cre	NA	Malignant gliomas	Zheng et al., 2008
	Nf1, Trp53, Pten	hGFAP-Cre	NA	Malignant gliomas	Chen et al., 2012
	K-Ras	BLBP-Cre	NA	Gliomatosis	Munoz et al., 2013
	Trp53, Nf1, and/or Pten	Nestin-CreER	NA	GBM	Alcantara Llaguno et al., 2015
OPC	PDGF	Retrovirus	NA	GBM	Assanah et al., 2006
	PDGF-B	RCAS/tv-a system	NA	O	Lindberg et al., 2009
	Pten, Trp53	Retrovirus + PDGF-IRES-Cre	Proneural	GBM	Lei et al., 2011
	TAZ, PDGFB	RCAS/N-tva system	Mesenchymal	Gliomas	Bhat et al., 2011
	NF1, PDGFA	RCAS/tv-a system	Mesenchymal	GBM	Ozawa et al., 2014
	PDGFB	RCAS/tv-a system	Mesenchymal	GBM	Ozawa et al., 2014
	Arf	RCAS/tv-a system	NA	A	Lindberg et al., 2014
	Ink4a, Arf	RCAS/tv-a system	NA	A	Lindberg et al., 2014
	Arf, PDGF-B	RCAS/tv-a system	NA	O	Lindberg et al., 2014
	Ink4a, Arf, PDGF-B	RCAS/tv-a system	NA	O	Lindberg et al., 2014
	Pten, Trp53	Retrovirus + PDGFB-IRES-Cre	Proneural	GBM	Lu et al., 2016
	Pten, Trp53, Olig2	Retrovirus + PDGFB-IRES-Cre	Classical	GBM	Lu et al., 2016
	Trp53	S100β-v-erbB	NA	O	Weiss et al., 2003
	ink/arf	S100β-v-erbB	NA	AO	Weiss et al., 2003
	Trp53	S100β-v-erbB	OPC like	O, GBM	Persson et al., 2010
	Trp53, NF1	NG2-Cre	Proneural	Malignant gliomas	Liu et al., 2011
	Trp53, NF1	NG2-CreER	Proneural	Malignant gliomas	Galvao et al., 2014
	Trp53, Nf1, and/or Pten	NG2-CreER	NA	Malignant gliomas	Alcantara Llaguno et al., 2015
Astrocyte	Ink4a, Arf, EGFR	Retrovirus	NA	High-grade gliomas	Bachoo et al., 2002
	Trp53, NF1	Lentivirus + GFAP-Cre mice	Mesenchymal	GBM	Friedmann-Morvinski et al., 2012
	Trp53, Pten	GFAP-CreER	Proneural, Neural, Mesenchymal	AA, GBM	Chow et al., 2011
	Trp53, Pten, Rb1	GFAP-CreER	Proneural, Neural, Mesenchymal	AA, AOA, GBM	Chow et al., 2011
	TgGZT_121_, Kras^G12D^, Pten	GFAP-CreER	Mesenchymal, Proneural, Neural	GBM	Vitucci et al., 2017
Neuron	Trp53, NF1	Lentivirus + Synapsin I-Cre or CamK2a-Cre mice	Mesenchymal	Malignant gliomas	Friedmann-Morvinski et al., 2012

*A, astrocytoma; O, oligodendroglioma; AA, anaplastic astrocytoma; AO, anaplastic oligodendroglioma; AOA, anaplastic oligoastrocytoma; GBM, glioblastoma multiforme*.

For instance, OPCs have been previously considered to mainly give rise to oligodendrogliomas and proneural subtype of GBMs (Weiss et al., 2003; Lei et al., 2011; Liu et al., 2011; Galvao et al., 2014). However, recent studies demonstrate that they can also serve as the cell of origin for astrocytoma (Lindberg et al., 2014) and other subtypes of GBMs, depending on the mutations initially introduced (Carro et al., 2009; Bhat et al., 2011, 2013; Lu et al., 2016). In particular, removal of Olig2 switches OPC-derived proneural subtype of GBMs into the classical subtype. Over-expression of TAZ or suppression of NF1, instead, readily induces OPC-derived GBMs into the mesenchymal subtype (Bhat et al., 2011; Ozawa et al., 2014). Similar observations were also obtained in astrocyte-originated GBMs, where the same GEMM can give rise to tumors with highly heterogeneous profiles (Chow et al., 2011; Schmid et al., 2016).

Importantly, the evolution routes of a defined cell of origin may also affect the molecular features of brain tumors. The recurrent GBMs from the same patients frequently switched their molecular features when compared to their primary tumor counterparts (Kim H. et al., 2015; Kim J. et al., 2015; Wang et al., 2016). Therefore, the molecular signature of a particular transformed tumor may not always reliably predict its cell of origin.

## Future Perspectives

Owing to genetic lineage tracing techniques and other advanced biological methods, tremendous progress has been made in understanding the glioma cell of origin during the past decade. Now, a consensus has been made that several important cell types, particularly NSCs and OPCs, are capable of transforming at least in GEMMs. However, many fundamental questions remain unanswered. For instance, is there a universal cell type functioning as the cell of origin for all gliomas in humans? Or alternatively, do different cell types give rise to gliomas with distinct pathological identities? Can different mutations drive the same cell of origin to follow the same or distinct routes toward the final transformation? When exactly do human gliomas form? GEMMs will surely continue to serve as the most important tools to address these fundamental questions. Nevertheless, we should be aware of the difference between GEMMs and patients. Newer methods and the concept of comparative pathology could help us identify what really initiates this devastating form of cancer in humans.

## Author Contributions

FS and CL wrote the manuscript. FS prepared the figure and the table.

## Conflict of Interest Statement

The authors declare that the research was conducted in the absence of any commercial or financial relationships that could be construed as a potential conflict of interest.
